# Targeted delivery of a cationic dendrimer with a plaque-homing peptide for the treatment of atherosclerosis

**DOI:** 10.1093/lifemedi/lnae039

**Published:** 2024-11-25

**Authors:** Tarik Zahr, Tianyu Li, Divya Bhansali, Qianfen Wan, Kam W Leong, Li Qiang

**Affiliations:** Department of Molecular Pharmacology and Therapeutics, Columbia University Medical Center, New York, NY 10032, USA; Naomi Berrie Diabetes Center, Columbia University Medical Center, New York, NY 10032, USA; Department of Biomedical Engineering, Columbia University, New York, NY 10027, USA; State Key Laboratory of Biochemical Engineering, Institute of Process Engineering, Chinese Academy of Sciences, Beijing 100190, China; Department of Biomedical Engineering, Columbia University, New York, NY 10027, USA; Naomi Berrie Diabetes Center, Columbia University Medical Center, New York, NY 10032, USA; Department of Medicine, Columbia University Medical Center, New York, NY 10032, USA; Department of Biomedical Engineering, Columbia University, New York, NY 10027, USA; Department of Systems Biology, Columbia University, New York, NY 10027, USA; Department of Pharmacology, School of Basic Medical Sciences; State Key Laboratory of Vascular Homeostasis and Remodeling, Peking University, Beijing 100191, China

**Dear Editor,**

Cardiovascular diseases (CVDs) lead to mortality across the globe, and atherosclerosis represents a major force in driving CVD-associated deaths. Atherosclerosis develops as fatty streaks accumulate along arterial walls, a process initiated by elevated levels of circulating cholesterol, particularly apolipoprotein B-containing lipoproteins. This excess cholesterol leads to the recruitment of macrophages, which engulf lipids, transform into foam cells, and deposit in the inner arterial lining [1]. Currently, lipid-lowering agents like statins and PCSK9 inhibitors are the primary drugs used to reduce the risk of atherosclerosis progression. However, atherosclerosis is increasingly appreciated as a chronic immune disorder ridden with plaque and systemic inflammation. Consequently, there is a growing interest in exploring anti-inflammatory or immunomodulating therapies, although these treatments pose the risk of systemic immunosuppression [1, 2].

Nanomedicine is emerging as an effective means of drug delivery and therapeutic intervention, thanks to the biocompatibility and physiochemical diversity of nanomaterials [2]. For example, polycation-based nanomaterials like polyamidoamine (PAMAM) dendrimers are known for their anti-inflammatory properties via their ability to neutralize negatively charged pathogens [3]. We have previously shown that P-G3, a third-generation PAMAM dendrimer with 32 surface amine groups, can preferentially deposit in the extracellular matrix (ECM) of white adipose tissue (WAT) when delivered intraperitoneally [4]. This accumulation is particularly noted in obesity, where the expansion of WAT and an increased amount of ECM results in an abundance of negatively charged biomacromolecules [5]. P-G3 effectively reduces inflammation and inhibits WAT expansion in such conditions [6]. These observations led us to explore the targeted delivery of P-G3 into atherosclerotic plaques, aiming for a noninvasive strategy to decrease the inflammatory burden within the plaque microenvironment.

We first hypothesized that P-G3, if administered intravenously, could accumulate in atherosclerotic plaques, either via macrophage uptake or through the leaky vasculature to adhere to the plaque matrix (Fig. 1A) [7]. P-G3 was efficiently taken up by cultured bone-marrow-derived macrophages (Fig. 1B), as well as by foam cells loaded with oxidized LDL (Fig. 1C). *In vivo* experiments involved administering Cy5-labeled P-G3 intravenously to *Ldlr*^*−/−*^ mice on a western diet with established atherosclerosis. Subsequent biodistribution analysis showed that while P-G3 was deposited primarily in the liver and kidneys, it was not present in the plaque-rich aorta at least when observed under low-resolution optical imaging (Fig. 1D).

**Figure 1.**
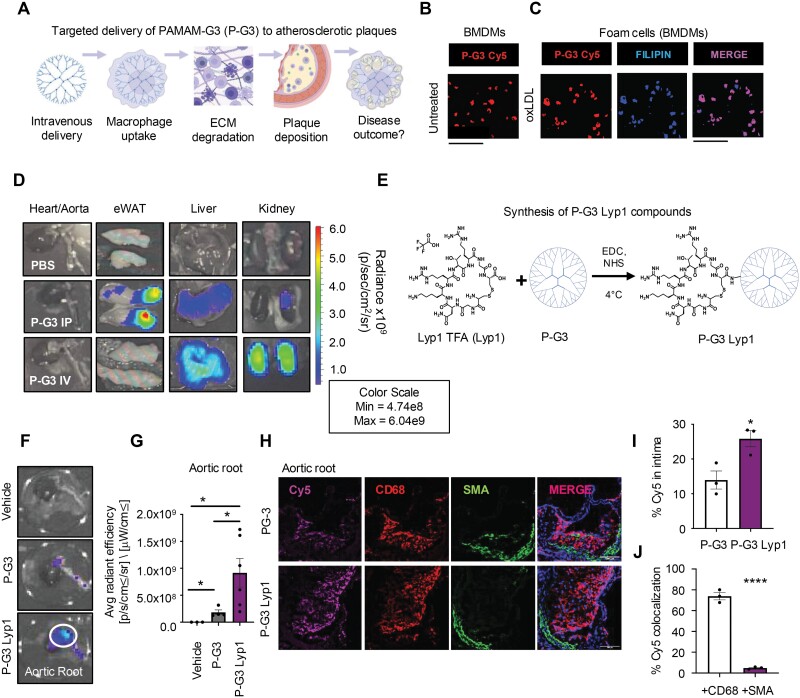
**Targeted delivery of P-G3 Lyp1 in atherosclerotic plaques.**(A) Schematic of our hypothesis illustrating PAMAM dendrimer delivery to ECM-rich atherosclerotic plaques via macrophage uptake or through leaky vasculature. (B) Bone-marrow-derived macrophages (BMDMs) were treated with 10 μg/mL Cy5-labeled P-G3 under basal conditions and (C) under oxidized LDL (oxLDL) loading to form foam cells. Filipin was used to detect intracellular free cholesterol. Cells were imaged at 40×, scale bar = 100 μm. (D) Tissue distribution of Cy5-labeled P-G3 dendrimers post-intraperitoneal (IP) or intravenous (IV) injection, visualized using IVIS optical imaging at 24 hours. (E) Structure of P-G3 Lyp1 compounds generated by conjugating a Lyp1 TFA plaque-homing peptide to P-G3 dendrimers. (F) Optical imaging of heart and aorta collected from mice 24-hour post-IV injection with 10 μg/g Cy5-labeled P-G3 or P-G3 Lyp1. (G) Quantification of radiance in the aortic root area circled in (F), calculated as average radiant efficiency, *n* = 3–6. (H) Confocal images of aortic root sections from mice injected with 10 μg/g Cy5-labeled P-G3 or P-G3 Lyp1, stained for CD68 and SMA and captured at 20×, scale bar = 100 µm. (I) Quantification of Cy5 fluorescence and (J) colocalization of Cy5 in + CD68 and + SMA areas, *n* = 3. Statistical significance was calculated using a two-tailed Student’s *t-*test for data points that passed normality with equal variances. *P < 0.05, ****P < 0.0001. Data are represented as mean ± SEM. Schematics were created with BioRender.com.

To enhance plaque targeting, we conjugated P-G3 with Lyp1 peptides (Fig. 1E). Lyp1 is a homing peptide that penetrates atherosclerotic plaques due to its recognition by p32 surface proteins found on macrophages [7, 8]. This modification significantly increased the delivery of P-G3 Lyp1 to the aortic root (Fig. 1F), achieving 5-fold higher concentrations than P-G3 alone (Fig. 1G). Cross-sectional analysis of aortic root leaflets confirmed enhanced accumulation of P-G3 Lyp1 within plaques (Fig. 1H and 1I), predominantly within intimal macrophages, as indicated by co-staining with CD68 (Fig. 1J).

Following the observed enhanced delivery, we treated atherosclerotic mice with P-G3 Lyp1 for 4 weeks to evaluate its impact on the plaque microenvironment and overall disease progression (Fig. 2A). P-G3 Lyp1 treatment significantly reduced total plaque area (Fig. 2B and 2C) and promoted a trend toward decreased plaque necrosis (Fig. 2D), but total cholesterol remained unchanged (Fig. 2E). Analysis of plaque inflammation revealed a substantial reduction in the total CD68-positive area with treatment (Fig. 2F and 2G). Notably, despite significant accumulation of P-G3 Lyp1 in the liver (Fig. 2H), no adverse effects were observed in assessing hepatic lipid content, liver fibrosis (Fig. 2I), or hepatoxicity, as measured by serum alanine aminotransferase (ALT) (Fig. 2J).

**Figure 2.**
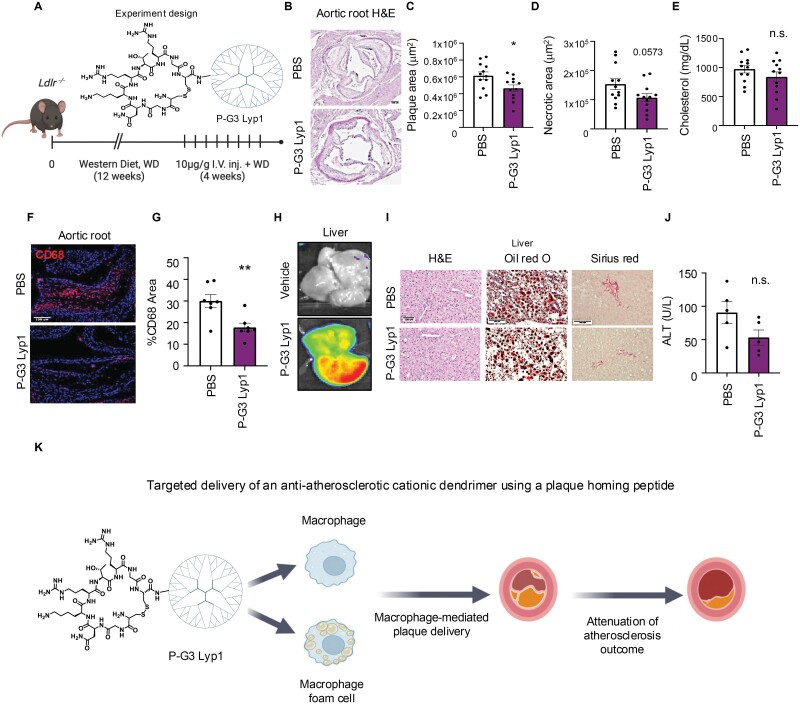
**Evaluation of P-G3 Lyp1 compounds for the treatment of atherosclerosis.**(A) Experimental design for an atherosclerosis study, where *Ldlr*^*−/−*^ mice were treated with 10 µg/g P-G3 Lyp1 for 4 weeks with western diet feeding, PBS was used as a vehicle. (B) H&E staining of aortic root sections, imaged at 4×, scale bar = 100 µm, to evaluate plaque morphometrics, like (C) total plaque area and (D) necrotic area, *n* = 12. (E) Total plasma cholesterol levels, *n* = 12. (F) CD68 staining of plaques to examine inflammation, imaged at 10×, scale bar = 100 µm, with (G) quantification, *n* = 7. (H) Optical image of liver collected from mice 24-hour post-IV injection with 10 µg/g Cy5-labeled P-G3 Lyp1. (I) Histological assessments of liver sections from atherosclerotic mice are described in (A). H&E staining at 10×, Oil Red O staining at 20× for lipid accumulation, and Sirius red staining at 20× for hepatic fibrosis, scale bar = 100 µm. (J) Serum measurements of ALT from mice described in (A), *n* = 5. (K) Graphical abstract of the study. Briefly, the anti-inflammatory cationic dendrimer PAMAM generation 3 (P-G3) is taken up by macrophages, and its conjugation with a plaque-homing peptide. Lyp1 allows for its delivery into atherosclerotic plaques to attenuate disease outcome. Statistical significance was calculated using a two-tailed Student’s *t-*test for data points that passed normality with equal variances. The plaque area was analyzed using the Mann–Whitney *U* test. *P < 0.05, **P < 0.01. Data are represented as mean ± SEM. Schematic was created with BioRender.com.

Taken together, these findings present the use of cationic nanomedicines, particularly those conjugated with a plaque-homing peptide such as Lyp1, as effective therapeutic and delivery agents targeting macrophage-ridden atherosclerotic plaques (Fig. 2K). Although previous studies have demonstrated the anti-inflammatory capabilities of PAMAM dendrimers in various conditions, their potential in atherosclerosis has not been explored. Our results support the role of Lyp1 in guiding PAMAM dendrimers to mitigate inflammation within plaques.

Future studies may explore expanding the scope of this targeted approach to consider other aspects of the plaque microenvironment, such as changes in ECM composition. This could offer mechanistic insights due to the preferential deposition of PAMAMs into the ECM. Lastly, integrating cholesterol-clearing agents into compound synthesis or in combination with our already proposed cationic dendrimers may accelerate the plaque-resolving effects observed in this report.

## Research limitations

Despite the abundance of P-G3 Lyp1 in plaques using our targeted delivery approach and the improvements found in atherosclerosis outcome with treatment, our study is limited by the phase of disease most relevant for compound efficiency. Whether P-G3 Lyp1 deposits in advanced plaques or more easily in the early stages of atherosclerosis will be explored in future studies, in addition to its role in potentially accelerating atherosclerosis regression.

The detailed methods are described in the Supplementary Data.

## Supplementary Material

lnae039_suppl_Supplementary_Material
