# Supplementary material for: Targeted delivery of a cationic dendrimer with a plaque-homing peptide for the treatment of atherosclerosis
Source: Life Med. 2024 Nov 25;3(6):lnae039. doi: 10.1093/lifemedi/lnae039 (PMC11761737; doi:10.1093/lifemedi/lnae039)
Supplement: lnae039_suppl_Supplementary_Material [file lnae039_suppl_supplementary_material.pdf]

Supplementary information

Targeted delivery of a cationic dendrimer with a plaque-homing peptide for the treatment of atherosclerosis

Tarik Zahr[1,2#], Tianyu Li[3,4#], Divya Bhansali[3], Qianfen Wan[2,5], Kam W. Leong[3,6*], Li Qiang[7*]

[1] Department of Molecular Pharmacology and Therapeutics, Columbia University Medical Center, New York, NY 10032

[2] Naomi Berrie Diabetes Center, Columbia University Medical Center, New York, NY 10032

[3] Department of Biomedical Engineering, Columbia University, New York, NY 10027

[4] State Key Laboratory of Biochemical Engineering, Institute of Process Engineering, Chinese Academy of Sciences, Beijing 100190, China

[5] Department of Medicine, Columbia University Medical Center, New York, NY 10032

[6] Department of Systems Biology, Columbia University, New York, NY 10027

[7] Department of Pharmacology, School of Basic Medical Sciences; State Key Laboratory of Vascular Homeostasis and Remodeling, Peking University, Beijing, China 100191

[#] Equal contribution from first authors

* Correspondence: Kam W. Leong, Li Qiang

Email: kwl2121@cumc.columbia.edu | qiang@pku.edu.cn

Methods

Mouse studies

All mice with a C57Bl/6J genetic background were housed at 22 ± 1 °C with a 12-hour light /12-hour dark cycle. Mice had access to food and water *ad libitum*. All experiments (except for macrophage isolation studies) utilized male *Ldlr*$^{-/-}$ mice (B6.129S7-Ldlrtm1Her/J, JAX, 002207). Atherosclerosis studies were conducted under western diet feeding (Inotiv, TD.88137, 42% kcal from total fat, and 0.2% total cholesterol) for 16 weeks (12 weeks of diet followed by 4 weeks of diet with twice weekly nanomaterial injections, n=12). Plasma cholesterol was measured with a Total Cholesterol E kit (Fujifilm Wako) using blood collected from left ventricular puncture of the heart in euthanized mice and separated via centrifugation. Serum Alanine Aminotransferase (ALT SGPT) was measured using a kit (Teco, A526-120). Experimental procedures were conducted under approval from Columbia University Medical Center's Institutional Animal Care and Use Committee (IACUC) and in accordance with the USDA Animal Welfare Act.

Morphometric analysis of the mouse aortic root

Mice were sacrificed after injection with nanomaterials using CO_2 euthanasia. Total body perfusion was done with saline after blood collection. The aorta was cleaned using butterfly scissors to remove perivascular fat and lymph nodes and dissected for embedding or optical imaging. Mouse hearts with the intact aorta were kept in PBS for optical imaging as described below. For plaque analyses, aortic roots were either collected and fixed in 10% formalin overnight followed by storage in 70% ethanol for paraffin embedding or embedded directly without fixation in molds with optimal cutting temperature (OCT) and frozen down. In both cases, aortic roots were sectioned (6 µm in thickness) in serial slices and onto charged frosted slides. Total plaque area and plaque necrosis were computed in Harris hematoxylin and eosin (H&E) sections, 60 µm apart, from start to finish of the aortic root leaflets, as described previously [1]. Images were taken on a

light microscope at 4X. Livers were also collected and stained with H&E, Oil Red O for lipid droplets, and Sirius Red for collagen deposition.

Synthesis of P-G3 Lyp1 compounds

P-G3 polymers were obtained from Dendritech. P-G3 (2.5 mg), EDC (3.3 mg, freshly prepared in 20 mg/mL water solution), and NHS (6 mg, freshly prepared in 20 mg/mL water solution) were mixed in PBS (use 10x PBS to get the proper 1x PBS concentration in the mixture). Lyp1 (0.4 mg, stored in 10 mg/mL water solution at -20 °C) was then added. The mixture (1-2 mL) was stirred (or shaken) at 4 °C overnight. The product was dialyzed for 24 hours to remove any unreacted compounds. For optical imaging experiments, P-G3 was conjugated with Cy5. P-G3 (10 mg) was mixed with Cy5-NHS (0.2-1 mg) in water (1 mL) and stirred (or shaken) overnight at room temperature (or 4 °C). The product was dialyzed to remove any unreacted Cy5-NHS. All polymers were injected at 10µg/g either once for optical imaging experiments or twice weekly for four weeks in the atherosclerosis treatment studies.

Optical imaging of Cy5-labeled P-G3 Lyp1

Tissue distribution of P-G3 and P-G3 Lyp1 was evaluated using a PerkinElmer IVIS Spectrum optical imaging system as described previously [2]. Briefly, both compounds were labeled with Cy5 and injected once 24 hours pre-sacrifice. Tissues are then collected after perfusion with saline, washed in PBS for four times, and kept in PBS upon the last wash before being subjected to imaging. Quantification of radiance was done using a Living Image 4.5.5 software and presented as the average radiant efficiency (n=3-6).

Bone Marrow-Derived Macrophage Isolation

Bone marrow-derived macrophages (BMDMs) were cultured as described previously [1]. Briefly, bones (femur and tibia) were collected from young adult mice and their bone marrow was flushed out via centrifugation. After red blood cell lysis and filtration, cells were seeded onto Petri dishes, cultured in DMEM (1g/L glucose, Corning, MT10014CM), and differentiated to macrophages

using 50 ng/ml macrophage colony stimulating factor (m-CSF, PeproTech) every 48 hours for one week. Upon differentiation, cells were seeded onto wells used for imaging, treated with 10µg/ml P-G3 overnight, and stained with Filipin to visualize cellular free cholesterol. Images were taken at 40X.

Immunohistochemistry

Slides containing aortic root sections of 6 microns in thickness were deparaffinized (for formalin-fixed, paraffin-embedded tissue) or fixed (for OCT-embedded tissue) and processed for immunohistochemical staining as described previously [3]. Primary antibodies used were anti-CD68 (BioRad, MCA1967GA), anti-SMA (Cell Signaling, D4K9N) at 1:200 overnight. Fluorescent secondary antibodies used were AF488 (Life Technologies, A21206), and AF568 (Life Technologies, AF11011) at 1:400 for 1 hour. Nuclear staining was done using 4′,6-diamidino-2-phenylindole (DAPI) at 1:10,000. Images were taken on a Zeiss confocal microscope with an LSM 710 scanning module at 10X or 20X.

Statistical analysis

Values are presented as the mean ± SEM. Data that fit the assumption of equal variances and normal distribution were assessed for statistical significance using an unpaired student *t*-test. Plaque analyses in the aortic root were evaluated using the Mann-Whitney *U* test and performed by the operator blinded to treatment group. Estimation of sample size, comprised of independent biological replicates, was based on previous studies of relevance and comparison. $P<0.05$ was considered statistically significant. Data was analyzed using GraphPad Prism version 10.0 (GraphPad Software, San Diego, CA).

Research ethics

Mouse experiments, including the isolation of bone marrow-derived macrophages for *in vitro* studies, were conducted under approval from Columbia University Medical Center’s Institutional Animal Care and Use Committee (IACUC) and in accordance with the USDA Animal Welfare Act.

Data availability

Details of the data and the materials used are provided in the main text. Any additional information is available upon reasonable request. Schematics were created with BioRender.com.

References

1. Zahr T, Liu L, Chan M, Zhou Q, Cai B, He Y, et al. PPARγ (Peroxisome Proliferator-Activated Receptor γ) Deacetylation Suppresses Aging-Associated Atherosclerosis and Hypercholesterolemia. Arterioscler Thromb Vasc Biol 2023;43:30–44. https://doi.org/10.1161/ATVBAHA.122.318061.
2. Wan Q, Huang B, Li T, Xiao Y, He Y, Du W, et al. Selective targeting of visceral adiposity by polycation nanomedicine. Nat Nanotechnol 2022;17:1311–21. https://doi.org/10.1038/s41565-022-01249-3.
3. Zahr T, Boda VK, Ge J, Yu L, Wu Z, Que J, et al. Small molecule conjugates with selective estrogen receptor β agonism promote anti-aging benefits in metabolism and skin recovery. Acta Pharm Sin B 2024. https://doi.org/10.1016/j.apsb.2024.01.014.
